# Examining physical activity and quality of life in adults with autism spectrum disorder and intellectual disability

**DOI:** 10.1177/17446295211033467

**Published:** 2021-09-13

**Authors:** Brianne Tomaszewski, Melissa N Savage, Kara Hume

**Affiliations:** TEACCH Autism Program, Department of Psychiatry, University of North Carolina at Chapel Hill, USA; The Department of Educational Psychology, University of North Texas, USA; School of Education, University of North Carolina at Chapel Hill, USA

**Keywords:** autism, quality of life, physical activity, adults, intellectual disability

## Abstract

Adults with autism and co-occurring intellectual disability engage in low levels of physical activity and are at increased risk of developing secondary health conditions attributed to physical inactivity compared to adults in the general population. Few studies have examined the use of objective measures to characterize physical activity levels for adults with autism and intellectual disability. The current study aimed to examine the relationship between physical activity, using an activity tracker, and quality of life in adults with autism and intellectual disability. In the current study, 38 adults with autism and intellectual disability, ages 18–55, wore a Fitbit Flex 2® activity tracker for 1 week, and completed the Quality of Life Questionnaire. The relationship between average daily step count quality of life was examined. Most adults in the sample were overweight and taking fewer daily steps than recommended guidelines. Increased average daily step count was significantly associated with quality of life.

## Introduction

There is a growing number of individuals with autism entering adulthood (Maenner et al., 2020). Autism is characterized by difficulties in social communication and restricted and repetitive patterns of behavior, according to the Diagnostic and Statistical Manual of Mental Disorders, 5th edition (DSM-5; American Psychiatric Association [APA] 2013). Symptoms must be present in early development and interfere with social, occupational, or other areas of functioning (APA 2013). Approximately 33% of individuals with autism have a co-occurring diagnosis of intellectual disability defined by an IQ score of below 70 (Maenner et al., 2020). Adults with autism and intellectual disability are at increased risk for developing health conditions such as obesity, hypertension, and diabetes in adulthood compared to the general population (Croen et al., 2015; Hildebrandt et al., 2010). An area of concern for the development of these health conditions is physical inactivity.

Physical inactivity is recognized as a severe public health problem (Kohl et al., 2012; Kruk, 2014). Diseases with increased risk for significant chronic health conditions due to physical activity include obesity, cardiovascular disease, cancer, stroke, and type 2 diabetes (Kallio et al., 2018; Kohl et al., 2012), with estimated costs attributed to physical inactivity ranging from 28 to 255 billion for adults living in the United States (Chenoweth and Leutzinger, 2006; Ding et al., 2016). Regular participation in physical activity is associated with a significantly reduced risk of chronic health conditions and mortality (Lee et al., 2012). Individuals with autism often do not engage in recommended physical activity levels (Sorensen and Zarrett, 2014). Physical activity engagement levels continue to drop for individuals with autism as they move into adulthood (Garcia-Pastor et al., 2019). Children and adolescents with autism are more likely to be overweight and be more physically inactive than typically developing adolescents (Broder-Fingert et al., 2014; McCoy and Morgan, 2020; McCoy et al., 2016).

There is emerging evidence for the benefits of physical activity participation in this population. In a study by Buchanan et al. (2017), parents reported that participation in physical activity had many benefits for their adult children, including healthy weight loss, reduced stereotyped behaviors, reduced anxiety symptoms, and improved social skills. Results added strong evidence that supports the importance of physical activity for physical and psychological benefits for individuals with autism reported in children and adolescents. (Bremer et al., 2016; Sorensen and Zarret, 2014; Stanish et al., 2015). Another area that may be relevant for adults with autism and intellectual disability is quality of life. The relationship between exercise and quality of life has been reported in both general and high-risk populations. Increased physical activity levels have been associated with increased quality of life in university students (Gill et al., 2013; Krzepota et al., 2015) and older working-age adults (Puciato et al., 2017; Rétsági et al., 2020). In addition, one study has reported a relationship between objective physical activity trackers to quality of life in adults attending behavioral health programs in Norway (Blom et al., 2019). These associations were present 15 months after exiting the program (Blom et al., 2020). These studies suggest that both higher self-report and objective physical activity levels are associated with higher perceived quality of life in adults.

The concept of quality of life is considered a relevant construct for public policy and the promotion of equal opportunities for individuals with intellectual and developmental disabilities (Verdugo et al., 2012). Components of quality of life include independence (personal development, self-determination), social participation (interpersonal relations, social inclusion, and rights), and well-being (emotional, physical, and material well-being) (Gómez et al., 2011; Schalock et al., 2016). Quality of life is considered a significant outcome in adulthood, and more research is needed to understand factors that influence quality of life in adults with autism.

There has been a growing body of research on quality of life in adults with autism (e.g., Ayres et al., 2018; Gal et al., 2015; Hong et al., 2016). Several studies including individuals with autism or their caregivers report experiencing a lower quality of life than individuals with intellectual disability, adults with psychiatric disorders (Arias et al., 2018; Barneveld et al., 2014), and typically developing children and adults (Kamio et al., 2013; Khanna et al., 2014; Lin, 2014; Lin and Huang, 2019). It is important to note that some studies have reported that quality of life in autism may be lower in these studies due to factors above and beyond an autism diagnosis (Arias et al., 2018). Studies that control for intellectual functioning and support needs have reported that group differences no longer exist (Arias et al., 2018; Totsika et al., 2010). However, when covariates were included, children and adults with autism and co-occurring intellectual disability had lower scores in quality of life domains related to social inclusion, interpersonal relationships, and physical well-being than typically developing children and adults (Arias et al., 2018). However, there are limitations in measuring this construct, including the use of more subjective measures (versus more objective measures used in tracking other adult outcomes like employment) and the reliance on informant ratings rather than self-report (Hong et al., 2016; Sheldrick et al., 2012). There are also limitations on what we know about what predicts quality of life in individuals with autism (Khanna et al., 2014; Knüppel et al., 2018; Mason et al., 2018; Moss et al., 2017; van Heijst and Geurts, 2015).

Few studies have examined the relationship between physical activity and quality of life in adults with autism and intellectual disability. One study reported that good physical health is a positive predictor of maternal reports of quality of life (Hong et al., 2016). However, physical health ratings were also maternal reports and rated as good/excellent or poor/fair. Self-reported physical activity has been reported as associated with health-related quality of life in young adults with and without autism (Hamm and Yun, 2019). While these studies provide preliminary evidence for the association between reported levels of physical health related to quality of life, to our knowledge, no studies have examined the impact of objective measures of physical activity in adults with autism. Current research also has not focused exclusively on adults with autism and intellectual disability. An objective measure of physical activity has been reported as having a stronger association with quality of life than subjective measures in typically developing adults (Anokye et al., 2012) and high-risk populations (Blom et al., 2019, 2020). These studies utilized wearable tracking technologies, ActiGraph accelerometers (ActiGraph, LLC, Pensacola, Florida, USA), including measures of the intensity level of physical activity and daily step counts. Increased daily step counts were associated with increased scores on quality of life measures (Blom et al., 2019). One study has reported a relationship between increased daily step counts using the Fitbit wearable activity tracker and quality of life (Yurkiewicz et al., 2018) in adolescents and young adults diagnosed with cancer. Findings from these studies suggest that daily step counts may be associated with quality of life. Daily step counts as a potential objective measure of physical activity, and associations with quality of life have not been examined in adults with autism and co-occurring intellectual disability.

### Current study

Despite the known health benefits of physical activity, few studies have adequately described this population’s physical activity levels and examined the relationship between objective measures of physical activity and quality of life. The purpose of this study was to (1) describe average physical activity levels for adults with autism and intellectual disability using wearable activity trackers for 1 week, (2) describe quality of life in adults with autism and intellectual disability, and (3) examine the relationship between step counts and quality of life.

## Methods

The study was reviewed and approved by the (removed for review) Institutional Review Board (# removed for review). The Institutional Review Board located at (removed for review) Office of Human Research Ethics is fully accredited by the Human Research Protection Program, Inc., which serves to protect the rights and welfare of human subjects. No study activities were completed without approval. The ethics board ensured that the protocols and procedures used in the study included informed and voluntary consent and assent of all participants. This study examines baseline data in an ongoing randomized controlled trial for adults with autism and intellectual disability, examining the effects of a 12-week self-managed exercise program on weekly step counts, quality of life, and body mass index (BMI).

### Participant inclusion criteria

Inclusion criteria for the present study included: (a) parent/caregiver report of a previous clinical diagnosis of autism from a licensed psychologist independent of the study (no formal documentation required); (b) score of 28 or higher on The Childhood Autism Rating Scale™–Second Edition (CARS™-2; Schopler et al., 2010) administered during the first in-person visit by a trained researcher; (c) IQ < 71 confirmed during the first in-person visit by a trained researcher administering the Leiter International Performance Scale-Third Edition to each participant (Leiter-3; Roid et al., 2013); (d) adult ≥ 18; (e) parent/caregiver report of no health conditions that may impact ability to increase step counts; and (f) willingness to wear the Fitbit during the first research visit. The CARS™-2 Questionnaire for Parents or Caregivers was also given to each participant’s caregiver to support direct observation findings.

### Recruitment

Participants were recruited from one southern and one eastern state within the United States. Recruitment occurred through local autism service agencies, autism support groups, autism research conferences, and online sharing of the website and flyer. For example, emails were sent to (names removed for review) asking professionals to pass along the study information (e.g., flyer, website) to families. The researchers also contacted transition programs/community agencies such as (names removed for review) to pass along the study flyer and recruitment website address to adults with autism and intellectual disability that attended their programs. Interested participants signed up through the website. The initial screening included a phone conversation between a member of the research team and the potential participant or the potential participant’s caregiver to determine potential eligibility and gather parent/caregiver report of a previous clinical diagnosis of autism. Following the initial screening, an in-person visit was arranged to confirm eligibility.

### Study procedures

Participants attended an eligibility visit and provided consent to participate in the study. If an adult participant with autism and intellectual disability was adjudicated legally incapacitated by a court decision, then the only party legally able to provide proxy consent is the legally authorized representative (LAR). For adult participants with autism and intellectual disability, we relied on participant consent if the participant was their own guardian and LAR consent if the adult participant was not their own guardian. In the cases where a participant had a LAR, an assent/consent form using an appropriate language level and graphics was used to ask participants with autism and intellectual disability participate in the study in addition to the LAR signing the assent/consent form. Adult participants with autism and intellectual disability who are their own legal guardian provided clear consent via written, oral, or communication through a speech generating device or low-tech communication aid to be included in the study. The research staff also relied on continued behavioral assent (e.g., a participant’s willing participate during eligibility and research visits).

Throughout the eligibility visit, a trained member of the research team gathered data for the CARS™-2 via direct observation. Nonverbal IQ was measured using the Leiter International Performance Scale-Third Edition (Leiter-3; Roid et al., 2013), administered to the adult with autism and intellectual disability. The Leiter-3 was selected as it does not require language skills and is designed for individuals with intellectual disability. Participant height and weight were measured to determine BMI and the Quality of Life Questionnaire (QOL-Q; Schalock and Keith, 1993) was used to measure perceived quality of life. Participants were introduced to the activity tracker and asked to wear the device for 7 days, starting the day after the eligibility visit. A member of the research team returned within 2 weeks to upload and record step counts. Acceptance was confirmed by the participant wearing the activity tracker during the eligibility visit and via caregiver report during the 7-day baseline phase and data recorded through the activity tracker’s application (app).

### Participant characteristics

Fifty-two participants were assessed for eligibility and 40 participants met inclusion criteria (N = 6 had a Nonverbal IQ > 70, N = 6 refused to wear the activity tracker). Two participants voluntarily withdrew from the study and 38 participants completed the study. Participants’ ages ranged from 18 to 55(Mean Chronological Age = 25.79 years, SD = 8.60 years), and approximately three-quarters of the sample were male (see Table 1 for participant characteristics).

**Table 1.** Participant characteristics (N = 38).

Characteristic	Mean (SD) or %
Age	25.8 (8.60)
Biological Sex (% Male)	71.1
Race	
African American	15.8
Arabic	2.6
Multi-Racial	13.2
White	68.4
Ethnicity	
Hispanic or Latino	11.1
Non-Hispanic or Latino	88.9
Household Income	
<$20,000	5.5
$20,000–$39,999	13.5
$40,000–$59,999	13.5
$60,000–$79,999	16.2
$80,000–$99,999	18.9
>$99,999	32.4
Co-Occurring Conditions*	
Anxiety Disorder	21.0
Attention Deficit Disorder/Hyperactivity	23.7
Depression	8.6
Type 2 Diabetes	7.9
Hypertension	5.2
Other	15.8
Nonverbal IQ	60.2 (10.3)
	Range 31–69

*Note.* *Percentages reflect the proportion of the sample that reported condition; some participants reported more than one condition.

### Measures

#### Demographics and health information

Parents or caregivers provided demographic information about the adult with autism and intellectual disability, including biological sex, race/ethnicity, household income, age of diagnosis with autism and who made the diagnosis, and additional co-occurring conditions. Parents or caregivers also provided information regarding the participant’s motor and fitness skills, including how often they exercise.

#### Quality of life

The QOL-Q is a 40-item questionnaire designed to assess the quality of life for individuals with intellectual disability. This measure has been used in two intervention studies in adults with and without intellectual disability and autism (Gal et al., 2015). The scale is administered in an interview format and assesses overall quality of life, consisting of scores from four subscales: satisfaction, competence/productivity, empowerment/independence, and social belonging/community integration. The satisfaction subscale includes 10 items related to general enjoyment, satisfaction with living arrangement, feelings of loneliness, and success. The competence/productivity domain includes 10 items related to educational or training programs. The empowerment/independent domain includes 10 items related to decision making and autonomy in daily life activities. The social belonging/community includes 10 items related to social activity participation and satisfaction, presence of friends, and how individuals are treated. If self-report is not appropriate (e.g., a participant does not provide responses), two raters who know the individual well answer the questionnaire. The average of the independent rater scores is used for the subscales and total score. Schalock and Keith (1993) documented the scale’s structural validity using factor analysis and reported adequate internal (Cronbach’s Alpha = .90), interrater (*r* = .83) and test-retest (*r* = .87) reliability as well as evidence of construct and concurrent validity. For this study, trained researchers assessed receptive and expressive language skills and administered the QOL-Q to potential participants capable of self-report. If a participant could not self-report, two individuals who knew the participant answered questions as proxies (Schalock and Keith, 1993). In the current sample, 86.8% of participants (N = 33) completed the self-report of the QOL-Q, and 13.2% of participants had proxy reporters (N = 5).

#### Physical activity

Participants wore the Fitbit Flex 2® activity tracker. The Fitbit Flex® is a valid device for measuring step counts (Diaz et al., 2015; Gomersall et al., 2016). The Fitbit Flex2® is a waterproof wristband tracker that can be worn during all daily activities (e.g., swimming). We instructed participants to wear the activity tracker during all waking hours for 1 week. Average steps per day were calculated from the total weekly steps. Step count categories included basal activity (<2,500 steps/day), limited activity (2,500 to 4,999 steps/day), low active (5,000 to 7,499 steps/day), somewhat active (7,500 to 9,999 steps/day), active (10,000 to 12,499 steps/day), and highly active (>12,500 steps/day; Tudor-Locke et al., 2009).

#### Body mass index

BMI was calculated as a participant’s weight in kilograms divided by his/her height in meters squared (kg/m^2^). Weight was measured using a Fitbit AriaÃ’ Smart Scale. BMI categories followed categories used by the Centers for Disease Control and Prevention. They included (a) underweight (BMI < 18.5 kg/m^2^), normal range (BMI 18.5 to 24.9 kg/m^2^), overweight (BMI 25 to 29.9 kg/m^2^), and obese (BMI ≥ 30 kg/m^2^) (Centers for Disease Control and Prevention, 2017).

### Data analysis plan

All data analyses were conducted in SPSS Version 26. Participants’ BMI and step counts were examined using descriptive statistics. A repeated-measures ANOVA was performed to compare within-group differences across the quality of life domains using the QOL-Q. Pearson’s *r* correlations were examined among objective physical activity measures (BMI, step count), quality of life using the QOL-Q, and age, and nonverbal IQ. A multiple linear regression was performed to examine the extent to which average daily step count, nonverbal IQ, and age were associated with quality of life.

## Results

### Physical activity characteristics

The average BMI for participants fell in the overweight category (Mean = 28.33, SD = 6.90) (see Table 2 for physical activity characteristics). Participant average weekly steps were in the *low active* range (Mean = 51,759 weekly steps or 7,394 steps per day, SD = 24,729 weekly steps or 3,533 steps per day). Parents and caregivers reported that most adults with autism and intellectual disability had typical motor skills, and they exercised 1–2 times a week.

**Table 2.** Physical activity characteristics.

Physical Activity Characteristic	%
**BMI Category**	
Underweight	0
Normal	39.5
Overweight	18.4
Obese	42.1
**Step Count**	
Basal Activity	0
Limited Activity	29.0
Low Active	23.8
Somewhat Active	36.8
Active	2.6
Highly Active	7.8
**Motor Skills**	
Poor	36.8
Typical	55.3
Advanced	7.9
**Times Exercise Per Week**	
0	10.5
1–2	50.0
3–4	29.0
5+	10.5

### Quality of life profile

A repeated-measures ANOVA was performed to examine the quality of life profile of the satisfaction, competence, social belonging, and independence domains on the QOL-Q. Overall, there were significant within-individual differences among the domains, F(3, 35) = 7.04, *p* = .001, η_p_^2^ = .38. Post-hoc paired samples *t-*tests were performed to examine between-domain differences using a Bonferonni correction of *p* < .008 for multiple comparisons. Adults with autism and intellectual disability or proxy reporters rated the satisfaction domain (M = 23.37, SD = 2.721) significantly higher than the competence domain (M = 18.89, SD = 6.75), t(37) = 4.18, *p* < .001, Cohen’s *d* = .67, the independence domain (M = 20.61, SD = 3.45), t(37) = 3.70, *p* = .001, Cohen’s *d* = .66, and the social belonging domain (M = 21.28, SD = 3.16), t(37) = 3.30, *p* = .002, Cohen’s *d* = .54.

### Correlations

Correlations were examined among quality of life and domains of quality of life, weekly step count, BMI, age, and nonverbal IQ using a Bonferonni correction of *p* < .006 for multiple comparisons. The quality of life total score was significantly associated with weekly step count (*r* = .56, *p* < .001). The quality of life competence domain was associated with weekly step count (*r* = .46, *p* = .004).

### Multiple linear regression

The total quality of life score was regressed onto average steps per day, nonverbal IQ, and age. Overall, the model was significant, F(3, 34) = 7.41, *p* = .001, R^2^ = .50, Adjusted R^2^ = .34. Average steps per day, nonverbal IQ, and age accounted for 34.2% of the variance in quality of life. Average steps per day significantly predicted quality of life (B = .000256, 95% CI .001, .004, *p* <. 001), controlling for nonverbal IQ and age. For every 1,000 increase in steps per day, the total quality of life score increases by 2.56 points. Nonverbal IQ also significantly predicted overall quality of life-controlling for average steps per day and age (B = .34, 95% CI .03, .66, *p* = .03). For every 1-point increase in nonverbal IQ score, the total quality of life score increases by .34 points. Age was not significantly associated with total quality of life, B = −.09 95% CI, = .08, .29, *p* = .68.

## Discussion

This study examined the physical activity of adults with autism and intellectual disability using an objective measure and the relationship between physical activity and self-report or proxy-reports of quality of life. Results of the study indicated that the current sample was predominately overweight or obese. On average, adults in the current study were taking approximately 7,000 steps per day, considered the low active range. The results examining the associations among physical activity and quality of life showed average daily step count as the strongest predictor of overall quality of life above and beyond nonverbal IQ and age, suggesting the importance of physical activity.

### Objective measure of physical activity

Activity trackers are becoming a popular tool for measuring activity to promote positive behavior change. More than 80% of the sample recruited for this study accepted the Fitbit Flex 2™ and wore the tracker for at least 7 days. This finding adds to the limited body of literature on the feasibility/acceptability of using FitbitÃ’ trackers to measure step counts for adults with ASD (LaLonde et al., 2014). While the research examining the performance of FitbitÃ’ trackers in free-living contexts supports using these devices for step counts, it cautions using them to examine physical activity intensity levels (e.g., time spent in moderate-to-vigorous activity; Diaz et al., 2015; Gomersall et al., 2016). Future research should consider using a reliable and valid tool if the purpose is to measure intensity levels of physical activity within free-living contexts for this population.

### Quality of life profile

Adults rated the satisfaction domain of the QOL-Q the highest and the competence/productivity domain the lowest. The satisfaction domain asks general questions about enjoying life (e.g., “how much fun and enjoyment do you get out of life”; Schalock and Keith, 1993), whereas the competence/productivity domain relies on the adult being employed. These findings are consistent with previous studies that report that adults generally rate their quality of life higher than employment outcomes (Hong et al., 2016; Moss et al., 2017). The competence/productivity domain reflects high unemployment rates in adults with ASD (Roux et al., 2015; Taylor and Seltzer, 2011). Both the independence/empowerment and social belonging/community domains were rated higher than competence, but these differences were not statistically significant. Previous studies have reported the most significant difficulties in social aspects of quality of life (Gal et al., 2015; Jennes-Coussens et al., 2006; Kamio et al., 2013; Kamp-Becker et al., 2010; Lin, 2014; Lin and Huang, 2019), but a majority of these studies focused on individuals with autism without co-occurring intellectual disability. Current findings may reflect a unique pattern of quality of life for adults with autism and intellectual disability and warrant further replication.

### Quality of life and physical activity

Higher average daily step count was significantly associated with a higher overall quality of life and the competence/productivity domain scores. One previous study in adults with autism showed a significant relationship between self-reports of physical activity and health-related quality of life measured by the World Health Organization Quality of Life scale (Hamm and Yun, 2019). This relationship is not surprising, given that this scale incorporates physical health as a domain and is defined as an individual’s subjective view of their health. This study also incorporated self-reported physical activity levels from adults identifying with autism. Their cognitive ability was not reported (Hamm and Yun, 2019). Current findings highlight the potential that physical activity is related to the quality of life in adults with autism and intellectual disability. To our knowledge, this is the first study to report a relationship with an objective measure of physical activity and in adults with autism and intellectual disability.

One potential explanation for the relationship between physical activity and competence/productivity domain on the QOL-Q is that it reflects employment and vocational skills. To our knowledge, previous studies have not examined these relationships in adults with autism and intellectual disability. Research in typically developing adults shows that engagement in physical activity is related to fewer work absences leading to increased work productivity (Losina et al., 2017). In adolescents and adults, physical activity is related to better quality relationships with peers (Lee et al., 2019; Smith, 2003). Based on the poor adult outcomes reported in adults with autism related to both employment and social participation (Howlin and Magiati, 2017), future studies should examine how increases in physical activity may promote these skills and lead to increases in quality of life.

This study explores the role and potential influence of objective physical activity on broader quality of life with this population. Physical activity may be conceptualized both as a valued outcome and a mediating variable that influences the relationship between independent variables and quality of life (Gómez et al., 2019; Schalock et al., 2016). Research in middle-aged and older adults showed that physical activity mediated the relationship between social and environmental factors and quality of life (Van Dyck et al., 2015), and research in adults with Multiple Sclerosis showed that physical activity mediated the relationship between functional disability and quality of life (Sung et al., 2013). These studies support that physical activity is a critical process that influences other adult populations’ quality of life outcomes. However, more research is needed to understand this complex relationship in adults with autism and intellectual disability. Age was also a non-significant predictor in the current study. This non-significance suggests that the relationship between physical activity and quality of life is significant across the ages in the study. However, given the wide range of ages participating in the study, future research should examine how age impacts the relationship between physical activity and quality of life. The current study provides the foundational empirical evidence for considering step counts as an important component of quality of life (Gómez et al., 2019).

### Limitations

There are several limitations to this study. First, this study is descriptive, does not examine the effects of our ongoing intervention or the full landscape of exercise opportunities for the participants, and has a small sample size that limits the current findings’ generalizability. However, given the paucity of research in this area, our limited understanding of this population, the potential for objective measurement tools, and possible links between step counts and QOL, the data are important to examine, and next replicate and expand. Next, the QOL-Q measure has not been validated for use with adults with autism. Future studies can work to utilize measures that have been validated with individuals with autism with intellectual disability. Third, the caregiver-reported diagnosis of autism was not confirmed with gold-standard tools such as the ADOS or the ADI-R. However, the severity of autism symptoms was determined through direct observation and caregiver questionnaire results using the CARS™-2. While this is not a diagnostic tool, the CARS-2 ST is recommended for use with individuals over the age of 6 with an estimated IQ of 79 or lower (Schopler et al., 2010). All participants were above the clinical cutoff of 28, which indicate levels of autistic behavior, with 21 (55%) in the mild-to-moderate autism symptom severity range and 17 in the severe range (45%).

## Conclusion

The needs for adults with autism and intellectual disability are vast, and most research and service options focus on accessing education, employment, housing, and a social network. However, the health needs of this population cannot be overlooked, and the findings from this study indicated that most adults with autism and intellectual disability were overweight and in the *somewhat active* or below ranges for physical activity. This indicates they are at higher risk for health issues and highlight the need for support and intervention for this population, focusing on increasing physical activity. This study also highlighted using a physical activity tracker as an objective physical activity assessment tool and the association between average daily step count and quality of life. Although more research is warranted to replicate these findings in larger samples to understand the relationship between physical activity and quality of life, targeted intervention programs are needed to support physical health and quality of life in adults with autism and intellectual disability.
